# Addiction is driven by excessive goal-directed drug choice under negative affect: translational critique of habit and compulsion theory

**DOI:** 10.1038/s41386-020-0600-8

**Published:** 2020-01-06

**Authors:** Lee Hogarth

**Affiliations:** School of Psychology, University of Exeter, Washington Singer Building, Perry Road, Exeter, EX4 4QG UK

**Keywords:** Motivation, Risk factors

## Abstract

Drug addiction may be a goal-directed choice driven by excessive drug value in negative affective states, a habit driven by strong stimulus−response associations, or a compulsion driven by insensitivity to costs imposed on drug seeking. Laboratory animal and human evidence for these three theories is evaluated. Excessive goal theory is supported by dependence severity being associated with greater drug choice/economic demand. Drug choice is demonstrably goal-directed (driven by the expected value of the drug) and can be augmented by stress/negative mood induction and withdrawal—effects amplified in those with psychiatric symptoms and drug use coping motives. Furthermore, psychiatric symptoms confer risk of dependence, and coping motives mediate this risk. Habit theory of addiction has weaker support. Habitual behaviour seen in drug-exposed animals often does not occur in complex decision scenarios, or where responding is rewarded, so habit is unlikely to explain most human addictive behaviour where these conditions apply. Furthermore, most human studies have not found greater propensity to habitual behaviour in drug users or as a function of dependence severity, and the minority that have can be explained by task disengagement producing impaired explicit contingency knowledge. Compulsion theory of addiction also has weak support. The persistence of punished drug seeking in animals is better explained by greater drug value (evinced by the association with economic demand) than by insensitivity to costs. Furthermore, human studies have provided weak evidence that propensity to discount cost imposed on drug seeking is associated with dependence severity. These data suggest that human addiction is primarily driven by excessive goal-directed drug choice under negative affect, and less by habit or compulsion. Addiction is pathological because negative states powerfully increase expected drug value acutely outweighing abstinence goals.

## Brief introduction to theories of addiction

Scientific and clinical theories seek to explain why addicts continue to take drugs despite experiencing consequential harms. This paper evaluates evidence for just three accounts of addiction—goal-directed choice under negative affect, habit and compulsion—to try and determine which mechanism plays the most important role in addiction. A brief summary of the broad scope of addiction theory follows [1], to place these three accounts in context.

Withdrawal-based negative reinforcement accounts argue that although euphoric drug effects maintain initial use, growth in the adverse withdrawal syndrome drives persistent drug use [2–4]. The self-medication account specifies psychiatric symptoms, which increase during abstinence, as the main driver of persistent drug use [5]. Although individual sensitivity to a multidimensional withdrawal syndrome and other negative states is associated with problematic substance use, there remains debate as to which component of withdrawal is most important [6], how this relates to psychiatric comorbidity [7, 8], and whether negative states prime drug seeking automatically [9] or via value-based decision making [10, 11]. This work forms the basis of the goal-directed choice under negative affect account evaluated at length later.

Positive reinforcement theories, by contrast, argue that the excessively rewarding effects of drugs drive persistent drug use independently of the withdrawal syndrome [12–15]. The challenge for positive reinforcement theories is to explain why drug use persists when addicts claim the drug has lost its value [16]. To solve this contradiction, a range of secondary processes have been postulated wherein drug seeking becomes less controlled. Theories that appeal to appetitive Pavlovian conditioning, for instance, argue that the pairing of drug cues with drug reward endows drug cues with capacity to elicit drug seeking, and sensitivity to this effect may underpin dependence [1, 17–20], because cue-reactivity is automatic [21], or because drug cues signal the accessibility (and hence greater utility) of the drug in the presence of drug cues [22, 23]. However, drug cue-reactivity is not reliably associated with dependence severity in humans [24–27], suggesting this mechanism probably does not underpin addiction.

Other positive reinforcement accounts have argued drug-seeking behaviour becomes involuntary (or ‘crystalized’ [28]) with practice. These theories argue that drug cues, or contexts, or the completion of a prior link in a drug seeking chain, elicit drug seeking ‘automatically’ in the sense of not being determined by drug craving [29], or ‘habitually’ in the sense of not being determined by an expectation of the current value of the drug [30], or ‘compulsively’ in the sense that costs associated with drug seeking are discounted and do not impinge on the behaviour [31, 32]. The habit and compulsion models are evaluated at length later.

Neurocognitive versions of positive reinforcement theory claim that although drug reward drives greater drug use, the persistence of this behaviour in addicted individuals is driven by acquired dysfunction in decision-making capacity. These accounts differ in focus. Addiction could be driven by global impairments in cognitive function [33], loss of volume/function of the prefrontal cortex and other brain regions [34], specific impairments in inhibitory control [35], or specific narrowing of temporal horizon such that future costs and benefits are not considered in decision making [36]. It remains unclear to what extent these neurocognitive dysfunctions can be methodologically isolated from each other, and whether they play a causal/prospective role in addiction or are non-functional consequences of drug exposure [37]. These neurocognitive models will not be considered further because there is insufficient space to do justice to this broad field.

The various theories for addiction are usually pitted against one other on the assumption that only one mechanism can explain addiction. However, multiple mechanisms could contribute simultaneously. Furthermore, the underpinning mechanisms could differ between individuals depending on developmental pathway, constitution, risk and protective factors [38, 39], or between drug classes, for example, stimulants vs. depressants [40], or across types of behaviour, for example, drug seeking vs. drug taking [41, 42]. However, methods do not exist to adequately isolate the contribution of specific mechanisms to behaviour in different conditions.

Box 1 defines the three theories of addiction evaluated in this paper, alongside key methods used to test each theory in laboratory animals and humans—providing a translational perspective. The methods used with each species are descriptively similar and arguably tap the same theoretical mechanisms, but multiple methodological differences make direct comparison between species challenging. Despite these complications, the weight of evidence does appear to provide converging translational support for the claim that addiction is primarily driven by excessive goal-directed drug choice under negative affect, and to a lesser degree by habit or compulsion.

Box 1: Definitions and methods of three addiction theories **Definitions**

Theory 1—Goal-directed drug choice under negative affect

On this view, individuals differ in their experience of drug reward value, due to a variety of constitutional risk factors. The frequency of goal-directed drug seeking is driven by an expectation of drug value (determined by experienced value) combined with knowledge of the voluntary behaviour necessary to obtain the drug [8, 15, 297–299]. The second process is that for some users, negative states (withdrawal, distress, pain, anxiety, depression etc.) are acutely mitigated by drugs, enabling these negative states to powerfully raise expected drug value (reflected in verbal ‘coping motives’), driving goal-directed drug seeking above already elevated baselines [10, 11], acutely outweighing competing abstinence goals [300].

Theory 2—Habit.

Drug seeking is initially goal-directed, but drug reinforcement progressively strengthens the association between drug stimuli (S) and the drug-seeking response (R), such that drug stimuli can elicit the drug-seeking response directly through an S−R association, without retrieving an expectation of drug value [30]. Addiction is driven by an increased contribution of this S−R/reinforcement mechanism to the control of drug seeking. Habit theory predicts that drug seeking is controlled by the established S−R strength only so long as the reinforcer is not re-experienced has having a different value. Once the reinforcer is experienced as having a lower or higher value, this will change the strength of the S−R association and the frequency of drug seeking will change accordingly (i.e. habitual drug seeking is flexible, but requires experience of the changed value of the drug to adapt).

Theory 3—Compulsion

Compulsion theory is akin to habit theory, except that the flexibility of drug seeking is argued to be lost. As with habit theory, drug seeking is argued to be goal-directed initially, and then transitions to become an S−R habit, but then in the third stage, becomes a compulsion—a maladaptive habit where the S−R association controlling drug seeking can no longer be modified by direct experience of the drug reinforcer [31, 32]. Because compulsive drug seeking is controlled by the established S−R association (and not by the S−R/reinforcement mechanism) drug seeking is not modified by direct experience of the low value of the reinforcer and so continues in perpetuity despite loss of value. This explains why drug seeking persists even though drug use is directly experienced as harmful, because this does not weaken the S−R association controlling drug seeking.

**Methods**

*Stress-induced reinstatement in laboratory animals*: In the reinstatement model, laboratory animals are first trained to self-administer an addictive drug. The response is then extinguished by omitting the drug, and responding declines. In the reinstatement test, animals are exposed to stress vs. no-stress (manipulated behaviourally or pharmacologically), and it is typically found that stress increases (reinstates) drug self-administration [83].

*Mood/stress-induced drug motivation in humans*: Human drug users are exposed to negative mood or stress induction via a range of methods (music, self-referential statements, mood congruent words, public speaking, cold pressor, heat pain, serial addition, video clips, guided imagery etc.), and contrasted to a no-induction control condition (either between-subjects or counterbalanced within-subjects). Drug motivation is measured post-induction via a range of methods (craving, choice, consumption, cognitive bias, economic demand etc.). It is typically found that drug motivation is increased in the induction vs. no-induction condition (sometimes also relative to pre-induction), demonstrating the ability of negative states to motivate drug seeking [7, 10, 301, 302].

*The outcome-devaluation task in laboratory animals*: Animals first learn an instrumental response to produce an appetitive reward. The response could be goal-directed (driven by an expectation of current reward value), or habitual (driven directly by an S−R association). To test this, the reward is devalued (in one group) by pairing it with lithium chloride-induced sickness or specific satiety (in a separate context so that the test context is not paired with the devaluation treatment). Then, at test, animals again have the opportunity to perform the instrumental response in extinction (so experience of the reinforcer cannot modify any S−R association controlling the response). The response is deemed goal-directed in being controlled by an expectation of the current value of the reward if the devalued group decreases responding in the extinction test. If they do not show a devaluation effect, the response is deemed habitual in being controlled directly by an S−R association. Four versions of this method have been used to test whether drugs promote habitual behaviour, as outlined in the text [30, 174–176].

*The outcome-devaluation task in humans*: Typically, participants learn that two responses (R1 and R2) earn different rewarding outcomes (O1 and O2). One outcome is then devalued by consumption to satiety or instructions that an outcome is no longer available. Finally, choice between the two responses is tested in nominal extinction (i.e. instructions that outcomes will not be signalled until the end). A decrease in responding for the devalued outcome suggests that the response is goal-directed in being controlled by an expectation of the current value of the outcome. But if responding for the devalued outcome does not decrease (relative to baseline, or non-devalued control group), then the response is deemed habitual in being elicited directly by an S−R association [111, 130, 199–203, 303].

*The two-stage task in humans*: In each trial, selecting one stimulus from the first-stage pair produces a ‘common’ and ‘rare’ second-stage pair with a 70:30 probability, respectively. If the other stimulus from the first-stage pair is selected, the probabilities of the second-stage pairs are reversed. Selecting a second-stage stimulus pays an amount that varies slowly over trials independently for each stimulus. Payoff is maximised by learning the transitional structure between stages and which second-stage stimulus currently pays most. The goal-directed (model-based) vs. habitual (model-free) status of responding is determined by the choice of first-stage stimulus following a trial where a stimulus from the rare second-stage pair paid most. Goal-directed participants will choose the other first-stage stimulus than they chose in the previous trial, giving a 70% chance of producing the same second-stage pair as the previous trial, to access the second-stage stimulus that paid most. By contrast, habitual participants will choose the same first-stage stimulus as they chose on the previous trial because that previous trial was reinforced, even though this choice gives only a 30% chance of producing the same second-stage pair as the previous trial. In short, the task measures whether participants make choices using knowledge of the transitional structure between stages, or simply repeat choices that paid off in the previous trial [229, 233, 278–281].

*Shock punishment of self-administration in laboratory animals*: Animals are first trained on a drug self-administration schedule (or seeking-taking chain, where an initial response is required to access the self-administration lever). After training, the self-administration response is punished by foot shock to quantify the decrease in responding relative to baseline and/or a no-punishment group. Compulsivity is indexed by less shock suppression of self-administration [60, 197, 283–295].

## Is addiction primarily driven by excessive goal-directed choice under negative affect?

### Studies with laboratory animals

This section will consider animal studies that test whether dependence vulnerability is due to greater expected drug value driving goal-directed drug seeking, particularly in negative states [43]. Drug value can be measured by giving laboratory animals a mutually exclusive choice between a response that earns the drug and a response that earns another reward such as food. The proportion of drug choices quantifies the relative value of the drug. Only some ‘vulnerable’ animals show preferential drug choice [44–46]. Drug choice can be increased by extended drug exposure [47, 48], and modified by manipulating the relative magnitude, delay or effort associated with the two rewards [49–59] and by the opportunity for social interaction [60, 61]. Thus, drug choice is modified by individual differences, and multiple decision parameters relevant to that choice.

The claim that animals make goal-directed choices between drug and food based on the expected relative value of the rewards is supported by two lines of evidence. First, rats that preferentially choose drug over food have a greater number of neurons in the orbitofrontal cortex (OFC), which selectively activate prior to performance of the drug choice, as if the ramping up of OFC neuronal activity is the genesis of the drug choice [62, 63]. The OFC also carries signals reflecting multiple dimensions of rewards such as magnitude, effort, delay etc., suggesting the OFC may be important in calculating the overall utility of rewards [64]. Finally, although there is a question of homology [65], the OFC plays a role in goal-directed decision making in humans [66], which may translate to animals.

The second line of evidence comes from the outcome-devaluation procedure used to determine whether behaviour is goal-directed [67] (see Box 1). In one study [68], rats were trained on a seeking-taking chain for cocaine before the taking lever was extinguished. This manipulation immediately reduced performance of the seeking response tested in extinction (in the absence of the taking lever), suggesting the seeking response was controlled by a goal-directed expectation of access to cocaine, rather than an S−R association. So although drug seeking can be goal-directed (see also [69–71]), as noted in the habit section, most animal studies suggest it is habitual (see also [72]). It remains unclear what the optimal parameters are for detecting goal-directed vs. habitual drug-seeking behaviour [73].

The most important question is whether negative states such as withdrawal and stress can motivate goal-directed drug seeking. The key study testing this prediction [74] found that when shifted to a state of heroin withdrawal, rats immediately increased their heroin seeking in extinction, suggesting withdrawal raised the expected value of heroin as a goal. Relatedly, other animal studies have demonstrated that withdrawal or conditioned withdrawal motivate drug vs. food choice, or reinstate drug self-administration, or activate negative emotional brain circuits. However, these motivational effects may not necessarily be goal-directed, there are several null effects to consider, and it is possible that the motivational impact of withdrawal may differ between drug classes [75–82].

The stress-induced reinstatement model has also produced mixed support for the goal-directed account (see Box 1). Behavioural and pharmacological stress induction procedures reliably increase single lever drug seeking and taking in the reinstatement model [83, 84]. Furthermore, sensitivity to this effect is increased by various ‘vulnerability’ factors: specifically, long vs. short access to drugs [85–88]; adolescent vs. adult onset of drug exposure [89, 90]; oestrous cycle [91]; individual heterogeneity [92]; protracted withdrawal [93]; and baseline anxiety level at test [94]. This work suggests that sensitivity to negative-affect-driven drug seeking may play a role in vulnerability to dependence [95]. However, it is not clear whether stress augments drug seeking by raising the expected value of the drug, or through a more automatic form of control [96]. Furthermore, a wide range of non-stress variables prompt reinstatement suggesting it may be a noisy index of emotional control of behaviour [97]. Finally, one study has found that yohimbine-induced stress did not increase drug over food choice (Ahmed et al., personal communication 2019). In sum, the studies reviewed here provide suggestive but preliminary evidence that drug seeking in laboratory animals can be goal-directed in some conditions, that withdrawal (and possibly stress) may motivate goal-directed drug seeking, and that individual variation in this motivational effect could underpin dependence vulnerability. However, more work is needed to convincingly determine that addiction in laboratory animals is driven by excessive goal-directed drug choice under negative affect.

### Human studies

This section will consider human evidence that drug dependence is associated with excessive goal-directed drug choice, especially under negative affect. Dependence symptom severity is consistently associated with greater economic demand (willingness to pay) for drugs, in both non-clinical [98–100] and clinical samples [101–103]. Economic drug demand also predicts treatment outcomes [99, 104], and drug consumption [105], and is increased by withdrawal [106, 107], stress induction [108], impulsivity [109], depression, anxiety [110] and schizophrenia [102]. To the extent that drug demand reflects *expected* drug value, these studies support the claim that goal-directed drug seeking increases with dependence and negative affect states (withdrawal, stress induction, psychiatric symptoms).

Excessive drug value indexed in human concurrent choice tasks is also associated with dependence. Participants make forced choices between drug and natural reinforcer over a series of trials. Different designs use points for rewards [111, 112], pictures of rewards [7, 113–117] or consumption of rewards [14, 118–124]. Preferential drug choice is reliably associated with the severity of dependence to heroin [125], cocaine [116, 117, 126, 127], alcohol [10, 15, 26, 112, 113, 115], and tobacco [15, 111, 114, 124, 128]. These associations have been found in both clinical [15, 113, 114, 116, 117, 125–127] and non-clinical samples [10, 26, 111–113, 115, 128]. Percent drug choice also increases with latency to relapse [129], abstinence [7], depression and anxiety symptoms and self-reported drinking to cope with negative affect [10, 15, 113, 115], and is decreased by health warnings and satiety [111, 130, 131], by raising the magnitude of the alternative reward [14, 112, 118, 121, 132–134], and by increasing the effort [59], and delay of the drug choice [112, 133, 134]. Thus, like economic demand, concurrent choice tasks index drug value, and this is increased in individuals with dependence and associated psychiatric risk factors.

The crucial question is whether drug choice is goal-directed as opposed to automatic. In support of the goal-directed account, drug choice is immediately reduced in extinction by decreasing the value of the drug via satiety [11, 111, 135], pharmacotherapy [130] and health warnings [111, 135], indicating that drug choice is goal-directed (see outcome-devaluation task in Box 1). The implication is that preferential drug choice in dependent individuals is controlled by greater expected drug value.

Crucially for the theoretical model, goal-directed drug choice has also been augmented by negative affect induction in two studies. In the first study, smokers were trained on a concurrent choice task to earn tobacco and food points, before tobacco was devalued by specific satiety [11]. Participants then completed either a negative or positive mood induction procedure before choice was measured again in extinction. For participants in the positive mood induction group, satiety decreased goal-directed tobacco choice as expected [111, 135]. By contrast, participants in the negative mood induction group who reported an increase in negative mood actually increased their goal-directed tobacco seeking, despite smoking satiety. The implication is that negative mood is a powerful motivational state driving goal-directed tobacco seeking that can outweigh the primary motivational state of satiety. In the second study, alcohol drinkers were trained on a concurrent choice task for alcohol and food points before being tested in extinction with a negative or positive affect statement read at random prior to each choice [10]. Negative affect statements primed an increase in goal-directed alcohol choice, relative to positive statements and baseline, in participants who reported drinking to cope with negative affect. The implication is that negative affect augments goal-directed drug choice, and this effect is magnified in those who are constitutionally predisposed to use drugs to cope with negative affect.

Other studies have demonstrated that, in vulnerable individuals, mood/stress induction has a greater priming effect on drug motivation measured by pictorial drug choice, craving, economic demand and consumption. The stress-induced increase in drug craving predicts risk of relapse in alcohol [136–139] and cocaine-dependent individuals [140, 141], suggesting this sensitivity is a core mechanism in addiction. Mood/stress-induced drug motivation is also amplified in individuals who self-report using drugs to cope with negative affect [10, 115, 125, 136, 137, 142–149], in smokers with depression symptoms [7, 150], young adult drinkers with depression symptoms [10] and alcohol-dependent men with anxiety symptoms [137]. Individual sensitivity to mood-induced drug seeking also correlates with withdrawal-induced drug seeking suggesting a common mechanism [7]. Importantly however, although alcohol dependence has been associated with greater mood/stress-induced drug motivation in some studies [147, 151–153], a sizable number of other studies have reported null associations [10, 113, 115, 137, 143, 146, 148, 149, 154]. The implication is that although dependence severity is associated with preferential goal-directed drug choice, negative affect-induced priming of drug motivation is predominantly linked to psychiatric symptoms and drug use coping motives, and this second process may represent the unique additional risk factor that drives addiction in those with psychiatric comorbidities and subclinical psychiatric symptoms.

Psychiatric symptoms, abuse/trauma history and associated drug use to cope with negative affect are major prospective risk factors for the development and persistence of drug dependence [155–173]. Furthermore, as shown in Table 1, self-reported drug use to cope with negative affect mediates the relationship between psychiatric/abuse/trauma severity and dependence severity, in a wide range of clinical and subclinical groups. Although the majority of studies listed in Table 1 are cross-sectional, precluding causal inferences, they nevertheless strongly support the hypothesis that drug dependence in a wide range of vulnerable groups is driven by excessive goal-directed drug choice under negative affect.

**Table 1:** Studies showing that self-reported drug use to cope with negative affect mediates the relationship between psychiatric/abuse/trauma symptoms and drug dependence severity.

Paper	Participants	Gender	Index of psychiatric/abuse/trauma symptom severity	Index of drug use to cope with negative affect	Index of drug/alcohol dependence severity
[249]	South African school-aged adolescents	Both	Childhood abuse in the Childhood Trauma Q	Adolescent Coping Orientation for Problem Experiences	Alcohol Use Disorder Identification Test
[250]	Canadian Aboriginal school-aged adolescents	Both	Hopelessness subscale of the Substance Use Risk Profile Scale	Drinking Motives Q	Bespoke excessive drinking questionnaire
[251]	Scottish school-aged adolescents	Both	Neighbourhood deprivation subscale of Scottish Index of Multiple Deprivation	Drinking Motives Q	Bespoke measure of drinking frequency
[252].	Armed service member reservists and veterans	Both	Psychological distress in the Kessler K-6 Q	Drinking Motives Q	Alcohol Use Disorders Identification Test
[253].	Veterans	Both	PTSD measured by DSM-V	Drinking Motives Q	Brief Young Adult Alcohol Consequences Q
[254]	University students	Both	Frequency of intimate partner violence	Drinking Motives Q	Rutgers Alcohol Problems Index
[255]	University students	Both	Childhood abuse in the Childhood Trauma Q	Marijuana Motives Measure	Marijuana Problems Scale
[256]	University students	Both	Neurotic personality: hopelessness and anxiety sensitivity subscales of the Substance Use Risk Profile Scale	Drinking Motives Q	Alcohol Use Disorder Identification Test
[257]	University students	Both	Avoidance subscale of the Liebowitz Social Anxiety Scale	Drinking Motives Q	Rutgers Alcohol Problem Index
[258]	Adult moderate drinkers	Both	Negative mood in Positive and Negative Affect Scales	Drinking Motives Q	Bespoke measure of acute alcohol use disorder symptoms
[259]	Prisoners with history of childhood sexual abuse	Female	Trauma symptoms checklist	Drinking Motives Q (modified for drugs)	Inventory of Drug Use Consequences
[260]	Adult community mental health clients	Both	PTSD Symptom Scale Interview version	Drinking Context Scale	First two items of Alcohol Use Disorders Identification Test
[261]	School-aged adolescents	Both	Bespoke bullying Q	Drinking Motives Q	Rutgers Alcohol Problem Index
[262]	University students	Both	Frequency of intimate partner sexual coercion	Drinking Motives Q	Rutgers Alcohol Problems Index
[263]	University students	Both	Beck Depression Inventory	Drinking Motives Q	Brief Young Adult Alcohol Consequences Q
[264]	University students	Both	Childhood abuse in the Childhood Trauma Q	Drinking Motives Q	Alcohol Use Disorder Identification Test
[265]	Homeless	Female	PTSD symptoms in Posttraumatic Diagnostic Scale	Drinking Motives Q	Timeline follow back of alcohol use frequency
[266]	University students with history of suicidal ideation	Both	The Adult Suicidal Ideation Q	Drinking Context Scale	Alcohol consumption in NIAAA Questions + Young Adult Alcohol Consequences Q
[267]	Sample from Virginia Adult Twin study	Both	Lifetime major depression in DSM-IV	Alcohol Use Inventory	Lifetime Alcohol dependence in DSM-IV
[268]	Prisoners	Female	Perceived Stress Scale	Substance Abuse Treatment Functionality Scale	Drug Abuse Screening Test
[269]	Woman exposed to domestic violent abuse	Female	Trauma Symptom Inventory	Drinking Motives Q	Timeline follow back: Number of heavy drinking days
[270]	Adult community members	Both	Depression symptoms in DSM-IV	Drinking Motives Q	Alcohol dependence symptoms in DSM-IV
[271]	Woman who have experienced adult sexual assault	Female	Bespoke lifetime trauma exposure Q	Two alcohol items from the Brief COPE Q	Bespoke problem drinking Q
[272]	Adult community members	Female	Single item endorsing childhood sexual assault vs. not	Drinking Motives Q	Bespoke alcohol dependence Q
[273]	Prisoners	Male	Antisocial behaviour in Psychopathy Checklist—Revised	Drinking Motives Q	Michigan Alcoholism Screening Test —Short
[167]	Adult community members	Both	Depression/anxiety symptoms	Drinking Motives Q	Bespoke drinking frequency and problems Q
[274]	University students	Both	Anxiety Sensitivity Index	Drinking Motives Q	Bespoke measure of drinking frequency
[275]	Adult community members	Female	Court-substantiated cases of child abuse and neglect vs. matched controls	Single item assessing use of alcohol to cope	Diagnosis of alcohol dependence
[276]	Adult community members	Both	Center for Epidemiologic Studies Depression Scale	Drinking Motives Q	Alcohol consumption frequency in the National Health and Leisure Time Survey
[277]	Adult regular gamblers	Both	Shame subscale of the State Shame and Guilt Scale	Coping subscale of the Gambling Motives Questionnaire	Problem Gambling Severity Index

This mediational role is observed in clinical and non-clinical samples, both genders, across age groups, countries, drug classes (including gambling), and with a wide range of questionnaires (Q). These studies suggest that excessive goal-directed drug seeking under negative affect (indexed by coping motive) drives addiction in vulnerable groups

## Is addiction driven by habit learning?

According to habit theory of addiction [30, 32], repeated experience of drug reward progressively strengthens the stimulus−response (S−R) association between drug stimuli and the drug seeking responses, such that drug stimuli become able to elicit drug seeking directly, without an expectation of the drug and its current value. Thus, drug seeking becomes less susceptible to voluntary control and decision making [30, 32]. The outcome-devaluation and two-stage procedures are the key sources of evidence for the habit account, and these studies are reviewed now.

### Studies with laboratory animals

Outcome-devaluation designs testing habit theory of addiction in animals fall into four categories. In the most compelling designs, animals learn that two responses earn drug and food in separate sessions. Then, in separate tests, each outcome is devalued and response rate for the devalued outcome is measured in extinction. Four such designs have found that the drug-seeking response does not decrease from baseline following devaluation, suggesting the behaviour is not goal-directed (controlled by expected outcome value), but is an S−R habit elicited by drug paired contextual stimuli [30, 174–176]. The food-seeking response, by contrast, is reduced by devaluation, indicating that it is goal-directed. These four studies provide the core empirical basis for the claim that drug seeking as opposed to natural reward seeking (in laboratory animals) is especially prone to habitual control.

In the second design, animals are chronically exposed to a drug (experimenter administered or consumed in the home cage), and then trained on a single lever for food. Food is then devalued, and the food-seeking response is tested in extinction. Eight such studies have shown that, at test, food seeking is insensitive to devaluation (habitual) in drug-exposed animals and sensitive to devaluation (goal-directed) in non-drug-exposed animals [69, 177–183], although three studies reported null group differences [184–186]. The implication is that chronic drug exposure renders reward seeking prone to habitual control, producing general behavioural autonomy. However, it is not clear how habitual natural reward seeking would lead to drug dependence.

In the third design, animals are trained on a single lever for drug, and sensitivity to devaluation is tested after minimal vs. extended training. Three studies have demonstrated that the drug-seeking response is initially goal-directed but then becomes habitual with extended training [69–71]. However, because food seeking also transitions from goal-directed to habitual control with training in animals [187] (not replicated in humans [188]), these findings do not inform us about the unique habit forming potential of drug seeking.

In the fourth design, animals are trained on a single lever for drug and tested for sensitivity to devaluation following a fixed amount of training. These studies have revealed drug seeking to be both goal-directed [68, 74], and habitual [189], so do not inform us about the unique habit forming potential of drug seeking.

There are two main criticisms of the animal outcome-devaluation model. First, habitual instrumental behaviour is generally only found when animals have access to a single lever in each session ([190, 191] but for one exception see [30]). By contrast, it is commonly found that when rats have concurrent access to two levers for different rewards in each session, drug seeking remains goal-directed [73], food seeking remains goal-directed despite chronic drug exposure [184, 192], and food seeking remains goal-directed despite extended training [193–196]. If one accepts that human drug users’ natural environment offers a multitude of responses for different rewards, then it must be concluded that habitual behaviour seen in the animal model has minimal ecological validity and likely does not play a major role in human addictive behaviour [197, 198].

The second criticism is that habitual control is fragile because sensitivity to devaluation is immediately restored in reacquisition tests where drug seeking produces the devalued reinforcer. This restoration of sensitivity to devaluation in reacquisition tests is found for both drug seeking [30, 174–176] and food seeking in chronically drug-exposed animals [180, 181]. If one accepts that in human drug users’ natural environment, extinction conditions rarely occur, but conditions comparable to reacquisition prevail (i.e. drug seeking is typically reinforced), then it must be concluded that the habitual behaviour seen exclusively in the extinction test of the animal model has limited ecological validity and likely does not play a major role in human addictive behaviour.

### Human studies

Table 2 summarises outcome-devaluation studies conducted with human drug users to test habit theory. There have been 11 tests published in 7 papers [111, 130, 199–203]. A scan of the ‘Support for habit theory’ column indicates that these tests yield an 8 to 3 ratio of evidence against habit theory. Additionally, the analysis below indicates that the three positive tests can be explained by general task disengagement producing impaired explicit knowledge of task contingencies, rather than a specific propensity to habit learning.

**Table 2:** Outcome-devaluation procedure testing habit theory of addiction.

1	2	3	4	5	6	7	8
Test #	Paper	Participants	Task design	Reduced devaluation in users vs. controls?	Reduced devaluation with dependence severity in user group?	Status of explicit contingency knowledge in drug user group	Support for habit theory
1	[111] expt. 1	Young adult smokers	Concurrent choice of tobacco and chocolate points. Devaluation by specific satiety.	—	No	Accurate	No
2	[111] expt. 2	Young adult smokers	Concurrent choice of tobacco and chocolate points. Devaluation by health warnings.	—	No	Accurate	No
3	[130]	Young adult smokers	Concurrent choice of tobacco and chocolate points. Tobacco devalued by 1 mg intranasal nicotine replacement therapy.	—	No	Accurate	No
4	[199]	Young adult smokers	Concurrent choice of water vs. chocolate points. Devaluation by specific satiety.	—	No	Accurate	No
5	[200] expt. 1	Treatment-seeking users vs. controls	Concurrent choice of water vs. crisp points. Devaluation by specific satiety.	No	—	Accurate	No
6	[200] expt. 2	Treatment-seeking users vs. controls	Concurrent choice of coca-cola vs. chocolate points. Devaluation by specific satiety.	No	—	Accurate	No
7	[203] task 2	Cocaine-dependent vs. controls	Concurrent choice of correct vs. incorrect avoidance response based on stimulus signalling a shock to left or right wrist. Devaluation by disconnecting one wrist.	No	—	Accurate	No
8	[201] task 2	Heavy smokers vs. controls	Comparable task to [203] task 2 above.	No	No	Accurate	No
9	[202]	Alcohol-dependent vs. controls	Fabulous fruit task.	Yes		Impaired S-R knowledge in users vs. controls^a^	Yes
10	[203] task 1	Cocaine-dependent vs. controls	Modified fabulous fruit task.	Yes	—	Impaired S-O, R-O and S-R knowledge in users vs. controls	Yes
11	[201] task 1	Heavy smokers vs. controls	Modified fabulous fruit task.	No	Yes	Not impaired in users vs. controls, but impaired S-R and S-O knowledge with dependence severity in users	Yes

Habit theory predicts reduced impact of devaluation on choice in drug users vs. controls, and/or as a function of dependence severity in the drug user groups, suggesting propensity to habit and/or impaired goal-directed control. Columns 5, 6 and 8 highlight that only 3 out of 11 tests supported the predictions of habit theory. Furthermore, column 7 shows a strong correspondence between impaired devaluation performance and impaired explicit contingency knowledge. In the three tests where devaluation performance was impaired (tests 9–11), explicit task contingency knowledge was also impaired, whereas in the eight tests where devaluation performance was intact (tests 1–8) explicit contingency knowledge was also intact, suggesting that the apparent evidence for habit may be due to impaired knowledge of task contingencies stemming from general task disengagement

^a^Note that in this paper, contingency knowledge data were not published originally, but are reported in the supplementary materials of the current paper

All the studies in Table 2 used an outcome-devaluation task in which there was a concurrent choice between two responses that earned different rewards. These rewards could be points for tobacco, food, soft drinks, or money plus a specific outcome picture, shock or aversive noise. Outcomes were then devalued by specific satiety, by instructing participants that outcome pictures would not earn money points, or by disconnecting the shock or noise. Finally, participants were tested for choice between responses in extinction. If participants show reduced choice of the response that led to the now devalued outcome, they are goal-directed, but if they do not reduce choice of the devalued outcome, they are habitual.

Habit theory predicts that the effect of devaluation on choice at test should be reduced in drug users vs. controls, and/or as a function of dependence severity in the user group, demonstrating a propensity to habit. Columns 5 and 6 of Table 2 summarise evidence for these predictions. Tests numbered 1–8 provide no evidence for habit theory, in that the devaluation effect was not reduced in the drug user group or as a function of dependence severity. This failure to support habit theory was found in both clinical (tests 5–8) and non-clinical samples (1–4). Furthermore, the failure to support habit theory cannot be attributed to the use of concurrent choice procedures (which tend to discourage habitual learning as noted earlier) because all of the tests in Table 2 used concurrent choice procedures, both those that failed (test 1–8) and those that notionally supported habit theory (test 9–11).

In those tests that supported habit theory (tests 9–11), the drug user group or more dependent users also showed impaired explicit knowledge of the contingencies operating in the task, in addition to weaker devaluation performance. Indeed, column 7 shows a perfect correspondence between impaired explicit contingency knowledge and impaired devaluation performance. In numerous human learning tasks, explicit knowledge of task contingencies is necessary for accurate performance, that is, participants who have impaired contingency knowledge perform less accurately in these tasks [204–209]. The implication is that drug users, or more dependent users, failed to acquire explicit contingency knowledge in the three tests supporting habit theory (tests 9–11), which impaired their devaluation performance, making them appear to be habitual. Drug users have general deficits in cognition or motivation that underpin their performance deficits in wide range of tasks [37, 210–212]. Arguably, this general deficit in cognition or motivation produced general task disengagement that impaired explicit contingency knowledge and thereby impaired devaluation performance. In other words, impaired devaluation performance is probably not driven by a specific propensity to habit learning or deficit in goal-directed control but by general task disengagement.

It is important to note that drug users’ deficit in explicit contingency knowledge extended to knowledge of stimulus−response (S−R), response−outcomes (R−O) and stimulus−outcome (S−O) contingencies. Thus, it cannot be claimed that drug users were specifically impaired in learning about outcomes important for goal-directed action, as has sometimes been claimed [201, 213]. For example, the supplemental material for test 10 [203] states that: “Compared with control volunteers, CUD [cocaine dependent participants] demonstrated significant deficits in explicit knowledge in terms of stimulus-outcome … response-outcome … and stimulus-response … relationships”. Similarly, in test 11 [201], “Strong evidence was obtained for a negative association between FTND scores [nicotine dependence severity in smokers] and explicit knowledge on stimulus–response…and stimulus–outcome associations”. Furthermore, hierarchical multiple regression analysis suggested that weaker knowledge of the stimulus–outcome contingencies explained the relationship between nicotine dependence severity and weaker devaluation performance, leading the authors to conclude that “habitual responding in severely dependent smokers may be the result of compromised goal-directed learning”. However, because this specific impairment in stimulus−outcome knowledge was not found consistently across the three studies supporting habit theory, this conclusion cannot be maintained. Indeed, test 9 [202] did not publish the explicit contingency knowledge data, but later analysis of these data (reported in the supplementary materials of the current paper) found less accurate explicit knowledge of stimulus−response contingencies in alcohol dependent vs. control participants—the very knowledge that should be important for habit learning. In sum, all three studies showing impaired devaluation performance also showed impaired explicit contingency knowledge suggesting that the apparent evidence for habit could be explained by general task disengagement perhaps driven by a general cognitive impairment [37, 210–212].

The idea that devaluation performance could be disrupted by general cognitive impairment is supported by ‘cognitive load’ studies in humans and animals. These studies have found that devaluation performance can be impaired by stress [214–219], acute alcohol administration [220], an alcohol consumption expectancy [135], being placed in drug-related contexts [221, 222], and sleep deprivation [223]. Furthermore, devaluation performance is impaired in a range of neuropsychiatric conditions including social anxiety [224, 225], autism spectrum disorder [225], schizophrenia [226], Parkinson’s disease [227], obsessive compulsive disorder [213], impulsivity [199], and in young children [228]. The generality of the devaluation deficit suggests it stems from general motivational or cognitive impairments, and is not the unique mechanism underpinning addiction.

A similar analysis may be applied to the two-stage task (see Box 1 for a description of the methods). The results of these studies are summarised in Table 3. There have been nine tests of whether model-based (goal-directed) learning is impaired or model-free (habit) learning is increased in drug users vs. controls (column 4), or as a function of dependence severity in the user group (column 5). Of the nine tests, only four claimed evidence for habit theory (although one of these was one-tailed and the group difference was not significant when a confound in cognitive speed was controlled [229]). Crucially, none of the studies measured participants’ explicit knowledge of the task contingencies. Consequently, it is unknown whether the four studies reporting evidence for habit theory can be explained by impaired contingency knowledge (as was the case with the outcome-devaluation procedure). However, it is known that model-based learning can be increased by adding incentives (points) for accurate performance [230], and impaired by a working memory load manipulation [231], and is impaired in individuals with lower working memory capacity [232], and lower cognitive speed [233, 234]. These findings suggest that the four two-stage studies that reported evidence for habit theory may be attributed to general motivational/cognitive deficit in drug users or as a function of dependence severity, rather than a specific propensity to habit learning or deficit in goal-directed control. To quote one two-stage paper [233]: “whether reduced model-based control in patients constitutes a disease-specific mechanism or results from general cognitive impairments can only be teased apart in future longitudinal studies”.

**Table 3:** Two-stage procedures testing habit theory of addiction.

1	2	3	4	5	6
Test #	Paper	Participants	Reduced model-based/increased model-free learning in users vs. controls	Reduced model-based/increased model-free learning with dependence severity in user group	Support for habit theory
1	[278] comparison 2	Currently abstinent alcohol-dependent vs. controls	No	No	No
2	[233]	Children of alcoholic fathers vs. controls	No	—	No
3	[279]	Alcohol-dependent vs. control	No	No	No
4	[280]	18-year-old social drinkers	—	No	No
5	[281] expt. 2	University students with substance use as a key measure	—	No	No
6	[229]	Alcohol-dependent vs. controls	Yes	—	Yes
7	[278] comparison 1	Methamphetamine dependent vs. controls	Yes	No	Yes/no
8	[282]	General online sample, with alcohol dependence severity as a key measure	—	Yes	Yes
9	[281] Expt. 1	University students with substance use as a key measure	—	Yes	Yes

Habit theory predicts reduced model-based or increased model-free learning in drug users vs. controls, or with dependence severity in the user groups, suggesting impaired goal-directed control/propensity to habit. Columns 4 and 5 highlight that only four out of nine tests have supported these predictions. Furthermore, one of the positive studies (test 6 [229]) was rendered null when the group difference in cognitive capacity was controlled. It remains unclear whether the other positive results stem from general motivational or cognitive deficits, or tap the prospective mechanism underpinning dependence

## Is addiction driven by compulsion (insensitivity to punishment)?

### Studies with laboratory animals

One of the major problems with habit theory noted earlier is that sensitivity to devaluation is restored immediately when responses produce the devalued reinforcer, so habit could not explain the persistence of drug seeking in the human natural environment. To negate this theoretical dilemma, it has been proposed that drug seeking is controlled by compulsion, defined as “a maladaptive stimulus-response habit” [31], and “as the maladaptive persistence of responding despite adverse consequences” [32] (it should be noted that other researchers use the term compulsion to mean a wide range of processes which are not considered here because they are difficult to test empirically [198]). The principal assay of compulsivity is the persistence of punished drug seeking. The problem, however, is that persistence of punished drug seeking can equally be explained by excessive value ascribed to the drug, outweighing the punisher [43]. Unique evidence for the compulsion model relies on demonstrating that persistence of punished drug seeking is not associated with excessive valuation of the drug in another assay. Early studies presented preliminary support for this dissociation between assays, but later studies that have employed more sensitive measures of drug value have indicated that persistence of punished drug seeking is associated with greater valuation of the drug, undermining the core behavioural evidence for compulsion theory.

Table 4 summarises studies that have measured the suppression of drug self-administration by shock punishment (the putative assay of compulsivity), and drug value in a second assay (e.g., self-administration frequency, breakpoints in progressive ratio tasks, persistence under extinction, and preferential choice of drug vs. natural reward). Studies reporting a dissociation between these two assays (column 5) support compulsion theory by suggesting that persistence under punishment cannot be explained by greater drug value. By contrast, studies reporting a correlation between these two assays contradict compulsion theory by suggesting that persistence under punishment may be due to greater drug value. Column 5 indicates that four studies support compulsion theory, and 11 studies support drug value theory. Overall, the evidence favours drug value over compulsion theory as an explanation for the persistence of punished drug seeking. One speculation is that vulnerable animals persist under punishment not because they are insensitive to costs, but because the punisher motivates drug self-administration in the following period to self-medicate. If this is true, then persistence of punished drug seeking could be another example of excessive goal-directed drug seeking under negative affect, not an example of compulsion.

**Table 4:** Studies that have tested compulsion theory of addiction by determining whether persistence of punished drug seeking is dissociated from drug value measured in another assay.

1	2	3	4	5
Test #	Paper	Criteria to identify vulnerable vs. nonvulnerable groups	Measure of drug value	Relation between assay of punishment suppression and drug value?
1	[283]	High impulsivity	Acquisition of single lever self-admin dose−response function	Dissociated
2	[284]	Long access	Acquisition and reacquisition of seeking-taking chain for single dose	Dissociated
3	[285]	N/A	Higher breakpoints for self-admin.	Dissociated
4	[286]	N/A	Higher breakpoints for self-admin.	Dissociated
5	[287]	Long access	Progressive ratio breakpoint	Correlated
6	[288]	High novelty preference	Progressive ratio breakpoint and persistence under extinction	Correlated
7	[289]	N/A	Economic demand measure akin to progressive ratio	Correlated
8	[290]	Long access	Acquisition and reacquisition of seeking-taking chain for single dose	Correlated
9	[291]	N/A	Economic demand measure akin to progressive ratio	Correlated
10	[292]	3 Crit model	Progressive ratio breakpoint and persistence under extinction	Correlated
11	[293]	3 Crit model	Progressive ratio breakpoint & persistence under extinction	Correlated
12	[197]	3 Crit model	Progressive ratio breakpoint and persistence under extinction	Correlated
13	[294]	3 Crit model	Progressive ratio breakpoint and persistence under extinction	Correlated
14	[60]	3 Crit model	Progressive ratio breakpoint and persistence under extinction	Correlated^a^
15	[295]	N/A	Choice of drug over natural reward	Correlated

The top four studies report a dissociation between these assays suggesting persistence of punished drug seeking has a different mechanism to drug value (or the design was not sensitive enough to detect the correlation). The bottom 11 studies reported a correlation between persistence of punished drug seeking and greater drug value, suggesting persistence may be driven by drug value outweighing costs. Overall, the evidence favours drug value over compulsion account. Several technical details of the studies are noteworthy. The majority of studies identified separate groups of animals as vulnerable vs. nonvulnerable to dependence using various criteria described in column 3 (e.g., impulsivity or the 3 crit model [296]). If vulnerable vs. nonvulnerable animals showed less punishment suppression but no difference in drug value, the measures were defined as dissociated. But if vulnerable vs. nonvulnerable animals showed less punishment suppression and greater drug value, the measures were defined as correlated (sometimes these studies also reported the correlation coefficient between punishment suppression and drug value, which corroborated the conclusion from the group contrasts). Other studies included a single group of animals (in which case column 3 was labelled as N/A), and reported the correlation coefficient between punishment suppression and drug value. Column 5 was labelled as ‘correlated’ if this relationship was positive and significant. Finally, column 4 labels the method used to measure drug value. There are a multitude of procedural parameters that could explain differential sensitivity of the measure of drug value between experiments

^a^Note there was no correlation between persistence of punished self-administration and choice of drug over social reinforcement in this case

### Human studies

Human studies designed to test whether dependence is associated with insensitivity to costs (cost discounting) have also provided minimal evidence for compulsion theory. Demand tasks measure the amount of drug participants would hypothetically consume across increasing prices (costs). The intensity of demand (maximum consumption at low price) is considered to be a relatively pure index of drug value unaffected by costs. By contrast, breakpoint—the price at which drug consumption drops to zero—is thought to be more sensitive to the impact of price costs on the decision to consume. Compulsion theory would be supported if dependence severity was more strongly associated with breakpoint than intensity, suggesting cost insensitivity is more important than drug value [235–237]. However, meta-analyses and systematic reviews of this literature have found that proxies for dependence correlate more consistently with measures of intensity than breakpoint [238–241], suggesting that dependence is more likely to be driven by greater drug value than cost discounting. However, one key study found that student drinkers with a family history of alcoholism were less sensitive to the effect of imagined next-day responsibilities on reducing alcohol demand [242], supporting the notion that dependence vulnerability may be linked to discounting costs imposed on alcohol. It remains to be seen what explains this discrepancy.

Deficits in reversal learning have been interpreted as evidence for greater cost discounting in addiction. In reversal learning tasks, participants learn that one response has a higher payoff than an alternative choice, before these response−reward contingencies are reversed. Drug users show deficits in reversal learning despite comparable acquisition of the initial contingencies [35, 243–245] (for similar findings with laboratory animals see [246, 247]). One interpretation is that drug users are less sensitive to the punishment of the incorrect choice, driving persistence of this choice. However the effect could also be due to impaired prediction error coding, cognitive inflexibility, or general task disengagement [35]. Reversal learning deficit therefore do not provide compelling evidence for cost discounting in addiction.

A recent study directly tested whether alcohol dependence was associated with discounting delay and opportunity costs imposed on alcohol seeking [112]. Student drinkers (*n* = 127, who varied in alcohol dependence symptom severity) made concurrent forced choices between alcohol and food points under conditions that manipulated the magnitude of points and the delay to receive points. Alcohol value was indexed by preferential choice of alcohol vs. food, whereas sensitivity to costs was indexed by the decrease in alcohol choice when food points were of greater magnitude (sensitivity to opportunity costs) and when alcohol points were delayed (sensitivity to delay costs). It was found that alcohol use disorder symptom severity was associated with increased alcohol choice indicating greater value of alcohol, but not with sensitivity to opportunity or delay costs imposed on the alcohol choice. This paper provided further evidence that dependence is driven by greater value ascribed to drugs, and not with greater discounting of costs imposed on drugs, i.e. compulsion theory was not supported.

## Summary and conclusion

The paper reviewed studies with laboratory animal and humans that tested whether addiction is driven by excessive goal-directed drug choice under negative affect, habit, or compulsion. There was substantial support for the first account, and limited support for the latter two. Animal studies supporting the excessive goal account found that drug choice was associated with dependence vulnerability, can be modulated by multiple decision parameters, is subserved by the OFC decision-making centre, and is goal-directed. However, there was only indirect suggestive evidence that negative states such as withdrawal and stress motivate drug seeking via a goal-directed mechanism, and that this effect might be amplified in vulnerable animals. This area needs more attention, back-translating human findings. Human studies, by contrast, supported the excessive goal account by demonstrating that economic drug demand increases with dependence, psychiatric symptoms, and stress induction. Similarly, concurrent drug choice is demonstrably goal-directed, is modulated by decision parameters, and increases with dependence, psychiatric symptoms, and mood/stress induction, and this latter effect is amplified in individuals who report psychiatric symptoms and drug use to cope with negative affect, and in those at greater risk of relapse. Finally, psychiatric symptoms, abuse/trauma history, and coping motives confer prospective risk of dependence, and coping motives mediate this risk. These data provide converging translational (and longitudinal) evidence that addiction is primarily driven by excessive goal-directed drug choice under negative affect, although evidential gaps do need to be addressed.

By contrast, the evidence for habit theory is weak, suggesting that this psychological process does not play a major role in human addiction. Only four animal studies showed that drug seeking is especially prone to habit, and although eight studies showed that drug exposure renders reward seeking habitual, it is unclear how this would drive dependence. Habitual behaviour is also abolished when animals are given a choice, and when responses are rewarded, and so could not explain human addictive behaviour in the natural environment where these conditions prevail. In humans, most studies found no evidence for habitual behaviour in drug users vs. controls, or as a function of dependence. The three studies supporting habit theory showed a correspondence between impaired devaluation performance and impaired explicit contingency knowledge. Furthermore, deficits in devaluation performance have been found with a wide range of ‘cognitive load’ manipulations and psychiatric symptom states, suggesting a general effect produced by task disengagement. Finally, only the minority of studies using two-stage tasks supported habit theory, and these could also be explained by task disengagement. Collectively the studies provide minimal evidence for a specific propensity to habit or impairment in goal-directed control as a major factor controlling human addiction.

The evidence for compulsion theory is also weak. In animals, the primary index of compulsivity—persistence of punished drug seeking in vulnerable animals—is most often associated with greater drug value indexed in a separate assay. The implication is that persistence of punished drug seeking is not due to insensitivity to costs imposed on drug seeking (compulsivity), but due to excessive value of the drug. Human studies have similarly found minimal evidence that dependence is associated with cost discounting in economic demand tasks, reversal learning tasks or concurrent choice tasks where costs are imposed on the drug choice.

The overall conclusion from this translational analysis is that addiction is primarily driven by excessive goal-directed drug choice under negative affect, and much less by habit or compulsion. This conclusion accords with other negative reinforcement models of addiction [3, 9, 248], except that in the current model, negative states enhance the expected value of the drug driving goal-directed drug choice [10, 11, 74], rather than priming drug seeking automatically as is commonly claimed [9]. Addiction is pathological not because it is automatic, but because negative states powerfully drive up expected drug value acutely outweighing other goals such as a job, abstinence, family and health, resulting in a return to drug use despite wishes to the contrary expressed at other times.

## Funding and disclosure

The authors declare that they do not have any conflicts of interest (financial or otherwise) related to the content of the paper. The research was supported by an Alcohol Change grant (RS17/03) and a Medical Research Council (UK, MRC) Confidence in Global Mental Health pump priming award (MC_PC_MR/R019991/1) to LH. Funders had no role in the study design, collection, analysis or interpretation of the data, writing the manuscript, or the decision to submit the paper for publication

## Supplementary information

Contingency knowledge in Sjoerds et al. (2013)
