# Supplementary material for: Addiction is driven by excessive goal-directed drug choice under negative affect: translational critique of habit and compulsion theory
Source: Neuropsychopharmacology. 2020 Jan 6;45(5):720–35. doi: 10.1038/s41386-020-0600-8 (PMC7265389; doi:10.1038/s41386-020-0600-8)
Supplement: Supplementary file 1 — Contingency knowledge in Sjoerds et al. (2013) [file 41386_2020_600_MOESM1_ESM.docx]

# Contingency knowledge in Sjoerds et al. (2013) [1].

Thanks to Sanne de Wit and Zsuzsika Sjoerds for access and help with analysis of these data pertaining to explicit knowledge of contingencies between stimuli responses and outcome pictures in Sjoerds et al. (2013) [1]. These data were not published in the original paper for methodological reasons explained below. To summarise the findings here: alcohol dependent participants were impaired in explicit knowledge of the S-R contingencies, supporting the claim that the deficit in devaluation performance may not have been due to a specific propensity to habit or impairment in goal-directed control, but general task disengagement stemming from motivational or cognitive weakness.

**Brief outline of tasks**

In each trial of initial instrumental discrimination learning, a stimulus picture signalled whether a left or right response would be rewarded with money points, which was accompanied by a specific outcome picture. Thus, left and right responses produced the same money points but different outcome pictures. Participants’ task was to earn money points, and outcome pictures were incidental to this objective. In the devaluation test, two outcome pictures were presented together, paired with each response. One picture was crossed through, and participants were told to choose the response associated with the uncrossed outcome picture, as only this response would be rewarded with money points. Accuracy in choosing the correct response (devaluation performance) indexed participants’ learning of the relationship between the two responses and the (incidental) outcome pictures they produced (i.e. goal-directed knowledge of response-outcome relationships). Alcohol dependent participants showed impaired devaluation performance compared to controls – the basis for claiming they are prone to habit.

Participants were then scanned (reported in Sjoerds et al. (2013) [1]) and completed two other tasks (not reported: stop-signal task and cue-reactivity), before completing a questionnaire testing explicit knowledge of the stimulus-response (S-R), response-outcomes (R-O) and stimulus-outcome (S-O) relationships operating in the training stage of the original task. There were two questions for each type of contingency, so participants could score 0, 1 or 2, where 1 is the chance level performance. The original paper did not report contingency knowledge data because the time delay between the task and the test of explicit contingency knowledge introduced an opportunity for memory decrement which could have masked or accentuated group differences.

Participants in the original study [1] were 42 individuals with alcohol use disorder (AUD) and 21 healthy controls (HC). Exclusion were applied in the original paper based on: (1) low quality imaging data; (2) responding on less than 90% of the trials during the instrumental learning task; (3) performance below chance level during the training phase of the instrumental learning task; and (4) urine tested positive for drugs (cocaine) or benzodiazepines directly prior to the assessments. The original paper thus analysed 31 AUD and 19 HC in. The current analysis applied these exclusions but further excluded 2 AUD participants because they did not complete the contingency knowledge questionnaire. Thus, in the current analysis was with 29 AUD and 19 HC.

**Results**

Figure 1 shows the contingency knowledge of alcohol dependent (AUD) and healthy control (HC) participants. ANOVA on these data yielded a main effect of knowledge type, *F*(2,92)=33.19, *p*<.001, *η_p_^2^*=.419, with significant differences between all knowledge types (*p*s<.001), indicating that S-R knowledge was the most accurate. However, there was no main effect of group, *F*(1,46)=2.63, *p*=.11, *η_p_^2^*=.054, or interaction between group and knowledge type *F*(2,92)=.56, *p*=.57, *η_p_^2^*=.012. When groups were compared on each knowledge type separately, there was a group difference for S-R knowledge, *F*(1,46)=4.90, *p*=.03, *η_p_^2^*=.096, and no group difference for R-O, *F*(1,46)=.43, *p*=.52, *η_p_^2^*=.009, or S-O knowledge, *F*(1,46)=1.49, *p*=.23, *η_p_^2^*=.032. These data suggest that the AUD group retained less explicit knowledge of the relationships between stimuli and responses from the training phase of the task. This finding may support the claim that AUD had generally disengaged from the task, which could account for their poorer devaluation performance without having to appeal to a selective propensity to habit or deficit in goal-directed control.


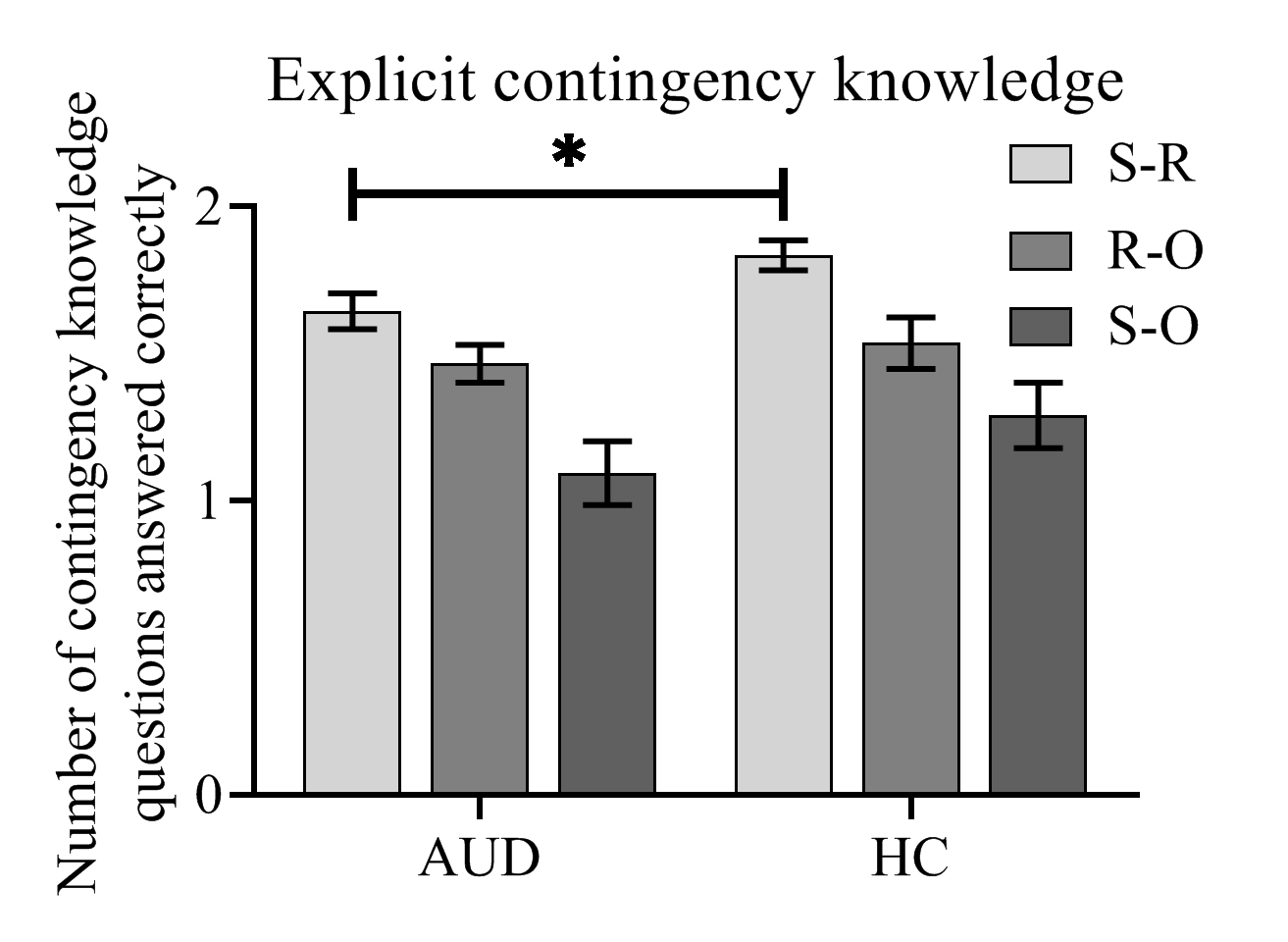
Figure 1. Explicit knowledge of stimulus-response (S-R), response-outcome (R-O) and stimulus-outcome (S-O) contingencies in healthy control (HC) and individuals with alcohol use disorder (AUD).

1 Sjoerds Z, de Wit S, van den Brink W, Robbins TW, Beekman AT, Penninx BW, et al. Behavioral and neuroimaging evidence for overreliance on habit learning in alcohol-dependent patients. Translational psychiatry. 2013;3:e337.
